# Letters on Syphilis

**Published:** 1852-02

**Authors:** 


### ECLECTIC DEPARTMENT,

### AND SPIRIT OF THE MEDICAL PERIODICAL PRESS.

---

*Letters on Syphilis, No.* 3.—By M. P. RICORD, of Paris. Translated by W. P. LATTIMORE, M. D., for the Med. Times.

*My Dear Confrere and Friend:*—The conclusion with which I closed my last letter: *That Blenorrhagia, the inoculated muco-pus of which gives rise to no result, does not recognize for its cause the syphilitic virus*—this conclusion, deduced from irrefragible facts, replaces the history of Blenorrhagia at the same point whence it was transmitted to us by the *Leviticus.* As old as man, yes, older than him, for animals created before him, are subject to Blenorrhagia, while they did not have syphilis, this disease, in its state of simplicity, has nothing in common with the syphilitic infection.

In spite of those who, since the time of Paracelsus, Bethencourt, and Fallopius, have wished to make a new disease of the Blenorrhagia non symptomatic of chancre, a disease identical with syphilis, the researches which I have made, corroborating the descriptions so precise, of Alexander Benedictus and of Cataneus, have given to the doctrines of Balfour, of Tode, and of Duncan, the value and solidity which Bell himself would have given them, had he been able, like us, to explain the pretended exceptional facts.

But Blenorrhagia—as I understand it, absolutely foreign to syphilis in its causes, in its form, in its consequences—does it depend upon a particular virus?

It would not be at all repugnant to admit a special cause, having the power specifically and constantly to produce Blenorrhagia and its consequences. In fact, nothing is more fitting to induce a Blenorrhagia than the muco-pus furnished by certain inflamed membranes.

But when we go back in the most rigorous manner, and with the severest criticisms, to the determining causes of the best characterized Blenorrhagia, we are forced to see and acknowledge that a Blenorrhagic virus is most usually wanting. Nothing is more common than to find women who have communicated Blenorrhagia the most intense, the most persistent, leading to *Blenorrhagic* consequences the most varied, and of the gravest character, who were only affected with uterine catarrhs, which were, sometimes, scarcely purulent. In other cases the menstrual flux seems to have been the only cause of the communicated disease. Finally, in a great number of cases we find nothing, or only simple deviations of diet, fatigues, excesses in sexual connections, the use of certain drinks — beer, of certain food — asparagus. From this arises that frequency of belief on the part of patients, a belief very often legitimate, that they owe their clap to a perfectly healthy woman.

On this point, I assuredly know all the causes of error, and I have the pretension to say, that no one more than myself, holds himself on his guard against frauds of every kind, scattered in the path of the observer; but it is with knowledge of the cause that I sustain this *proposition*. WOMEN FREQUENTLY GIVE BLENORRHAGIA, WITHOUT HAVING IT. Blenorrhagia, such as some persist in understanding it, (that is to say, as a consequence of contagion,) is as rare in woman as it is frequent in man. I do not think I go too far in saying that women give twenty claps for one which they receive. And this is easily understood, for women, so subject to discharges from the genital organs, which one cannot attribute to syphilitic causes, are the most frequent source of discharges in men which can be attributed to contagion.

It is impossible for me to take as serious, the doctrine of my learned colleague, M. Cazenave, who acknowledges that many women, under the influence of chronic utero-vaginal catarrhs, could have sexual relations without communicating anything, provided they were not heated to boiling point; provided they were not raised, so to speak, to a virulent red heat.

Is it not more simple to understand, and more rational to say, that with a less degree of excitation, the secretions are less irritant, and that custom can produce an immunity from these secretions in some persons, as it were a kind of acclimation.

It is thus, as I have frequently seen, that a married woman may cohabit with her husband without communicating anything to him; but let a lover supervene, and this last contracts a Blenorrhagia. The husband was acclimated, the lover was not.

When one studies Blenorrhagia without prejudice, without preconceived ideas, he is forced to confess, that it is frequently produced under the influence of most of the causes which determine inflammations of other mucous membranes.

The experience of Swediaur is at hand to prove this. This observer injected a volatile alkali into the urethra, and it produced a Blenorrhagia. Does this experiment prove that we can always, and at will, produce Blenorrhagia, by irritating injections. No; no more than one can always produce a Coryza by the same means, an Ophthalmia, etc. For Blenorrhagia, as for any other inflammation, there is need of a pre-existing predisposition, that immense unknown which governs all pathology. What proves this is, that Blenorrhagia is not always contracted under the same conditions where it is most evidently communicable. Without this happy immunity which the absence of the predisposition gives, Blenorrhagia, already very common, would be still more so.

An experience of twenty years has taught me to affirm, that excepting

Blenorrhoidal discharges — symptomatic of chancre, it is often perfectly impossible to recognize the cause of a discharge.

I am aware that many of my colleagues obstinately refuse to admit this opinion; in presence of every Blenorrhagia, they are preoccupied with syphilis, and their therapeutical prescriptions are only the logical consequence of this preoccupation.

Here, my dear friend, I ought to make you a confession, and I will make it publicly. This persistence of some of my honored and learned colleagues in always considering and treating Blenorrhagia as an accident of a syphilitic nature, has many times moved me. Thus it has frequently happened to me, not to satisfy a frivolous curiosity, much less to yield to a culpable incitement to aspersion, but to enlighten and assure my understanding; frequently, I say, it has happened to me to have recourse to a stratagem, of which I wish to make the avowal with all the reserve and propriety which I owe to the honorable confreres.

It has occurred under the following circumstances: a man presented himself, at my consultation, with one of the best characterized Blenorrhagias. He declared to me that he had only had connection with one woman, and that this woman was his wife, or mistress. This man was alarmed and disquieted. He brought with him the woman from whom he had contracted his disease, and she, protesting her innocence, in concert with the patient, supplicated me to submit her to the most rigorous examination. This examination, made with all the rigor and attention of which I am capable, showed me the sexual organs of this woman to be perfectly healthy. Nothing, absolutely nothing, in the deepest folds of these organs, which could explain the Blenorrhagia of this man. I requested the woman to step into another room, and, alone with the patient, I exhausted all possible means, the details of which I will spare you, to arrive at this certainty. The patient had only had connection with this woman; it was only in this intercourse that he could have contracted this disease.

I reassured the husband, or lover; I exculpated the wife or mistress; but then I asked them to become accomplices of the little stratagem which I am about to indicate.

I sent them both (separately, of course,) to this or that learned colleague, whom I knew to be in absolute opposition to me on the question of Blenorrhagia. I said to the patient: ask this question distinctly, Is my Blenorrhagia syphilitic? I said to the woman: ask boldly, Can I give Blenorrhagia to a man?

The couple returns: the man with a diagnosis thus arranged: *Syphilitic Blenorrhagia*, follow the treatment *ad hoc;* the woman had this indication: *the perfectly healthy state of the genital organs permits me to declare that Madame cannot have communicated a disease which she has not.*

It is not an unique and isolated fact which I cite to you, my dear friend; this experiment I have renewed frequently, and often enough to corroborate my convictions, and to assure my conscience.

What do these facts signify? That the cause of Blenorrhagia cannot always be known; that this malady may be produced by the causes common to all inflammations, provided there be a predisposition to it; but that the most special agent of Blenorrhagia is the muco-pus furnished by the inflamed genito-urinary mucous membranes.

This view of the case seems to me more rational, much more philosophical than that which would attach the Blenorrhagia, called venereal, to a kind of demivirus imagined by our very learned confrere and skillful syphilographist, M. Baumes. According to him, Blenorrhagia is, as it were, a degenerescence of chancre; it may give rise to a constitutional syphilitic infection, more feeble, however, than that produced by chancre, but without power, nevertheless, by contagion or inoculation, to produce this last. "We can then predict," adds M. Baumes, "the greatest similitude between the constitutional symptoms which are the result of one and the other of these diseases; and, in fact, experience proves that the difference between these symptoms lies, not in their nature, but only in their degree of intensity, in their gravity, and in their seat, which, after Blenorrhagia, usually extends to fewer tissues, to less numerous organs, than after chancre." (Baumes, *A Theoretical and Practical Treatise of Venereal Diseases*, vol. I, p. 259.)

That is veritably a doctrine of the golden mean. This pure theory is justified neither by facts, nor observation, nor experience; it wants only one condition — proof.

Thus far, then, and this is really my opinion, simple Blenorrhagia remains completely foreign to syphilis, so far as relates to the causes which produce it.

But in objection it is said, the pus of chancre, that is to say the syphilitic views, can produce Blenorrhagia. This opinion is very ancient; it has been sustained since the first appearance of the pox in England, and very beautifully can it be sustained now. But, what does this say? Does one depend on the observations of the ancients? They are incomplete and insufficient; it is impossible with them to proceed scientifically from cause to effect. Would one make experiments similar to those of Harrison, who believed in the production of Blenorrhagia from the introduction into the urethra of the pus furnished by a chancre, without knowing what it had physically produced? No! but more simply and more logically, we will conclude on the possibility of the production of a non-virulent Blenorrhagia by the pus of a chancre, in considering this pus, as had been done before me, as acting in the manner of a simple irritant. A woman having chancre at the inoculable period, may thus determine in a man, a Blenorrhagia which will not inoculate. We may thus explain the observations of Swediaur and others, supposing they made no error in diagnosis, seeing that they used neither the speculum nor inoculation; observations proving that men affected with chancres have communicated Blenorrhagia to women.

Here is what clinical observation teaches, and what experimentation can demonstrate. It is not rare to see patients, who, at first affected with a chancre of the glans or prepuce, are successively seized with a balanitis or a balano-posthitis determined by the irritating action of the pus of the chancre. But then, while the chancre furnishes inoculable pus, the balano-posthitis does not; (hereafter we will see, that in order that the pus of chancre may act specifically, conditions are necessary which are not always present.)

Adhering, then, to my first conclusion, reducing to their just value these first objections, I affirm that when Harrison produced Blenorrhagia with the pus of chancres, this pus either acted in the manner of simple irritants, or it produced an urethral chancre; this he did not verify. In the same way we will see that when Hunter produced a chancre with the pretended pus of a Blenorrhagia, it was with the product of a veritable urethral chancre that he had to do.

But if inoculation has proved that the cause, or causes of Blenorrhagia, *whatever be its seat* in the two sexes, differs from the specific cause, from the virus which *infallibly* produces chancre, the consequences then, of Blenorrhagia ought always to differ from those of chancre; and yet how many cases of constitutional syphilis, are attributed to Blenorrhagia.

These are questions, my dear friend, which will form the subject of my next letter. We will then see if it be possible to establish a differential diagnosis between affections which some wish systematically to confound.

You will first permit me to say a word of the incubation of Blenorrhagia.

Yours, RICORD.

